# Supplementary material for: Atherosclerotic plaque development in mice is enhanced by myeloid ZEB1 downregulation
Source: Nat Commun. 2023 Dec 14;14:8316. doi: 10.1038/s41467-023-43896-7 (PMC10721632; doi:10.1038/s41467-023-43896-7)
Supplement: Supplementary file 1 — Supplementary Information [file 41467_2023_43896_MOESM1_ESM.pdf]

SUPPLEMENTAL INFORMATION

FOR

Increased Lipid Accumulation in Macrophages in Response to ZEB1 Downregulation

MC Martinez-Campanario[1], Marlies Cortés[1#], Alazne Moreno-Lanceta[2#], Lu Han[1], Chiara Ninfali[1], Verónica Domínguez[3], María J Andrés-Manzano[4,5], Marta Farràs[6,7], Anna Esteve-Codina[8], Carlos Enrich[2,9], Francisco J. Díaz-Crespo[10], Belén Pintado[3], Joan C. Escolà-Gil[6,7], Pablo García de Frutos[5,11,12], Vicente Andrés[4,5¶], Pedro Melgar-Lesmes[2,13,14,15¶], Antonio Postigo[1,14,16,17*]

[1] Group of Gene Regulation in Stem Cells, Cell Plasticity, Differentiation, and Cancer, IDIBAPS, 08036 Barcelona, Spain
[2] Dept. of Biomedicine, University of Barcelona School of Medicine, 08036 Barcelona, Spain
[3] Transgenesis Facility, National Center of Biotechnology (CNB) and Center for Molecular Biology Severo Ochoa (UAM-CBMSO), Spanish National Research Council (CSIC) and Autonomous University of Madrid (UAM), Cantoblanco, 28049 Madrid, Spain
[4] Group of Molecular and Genetic Cardiovascular Pathophysiology, Spanish National Center for Cardiovascular Research (CNIC), 28029 Madrid, Spain
[5] Center for Biomedical, Research Network in Cardiovascular Diseases (CIBERCV), Carlos III Health Institute, 28029, Madrid, Spain
[6] Dept. of Biochemistry and Molecular Biology, Institute of Biomedical Research Sant Pau, University Autonomous of Barcelona, 08041 Barcelona, Spain
[7] Center for Biomedical Research Network in Diabetes and Associated Metabolic Diseases (CIBERDEM), Carlos III Health Institute, 28029 Madrid, Spain
[8] National Center for Genomics Analysis (CNAG), 08028 Barcelona, Spain
[9] Group of signal transduction, intracellular compartments and cancer, IDIBAPS, 08036 Barcelona, Spain
[10] Dept. of Pathology, Hospital General Universitario Gregorio Marañón, 28007 Madrid, Spain
[11] Dept. Of Cell Death and Proliferation, Institute for Biomedical Research of Barcelona (IIBB), Spanish National Research Council (CSIC), 08036 Barcelona.
[12] Group of Hemotherapy and Hemostasis, IDIBAPS, 08036 Barcelona, Spain.
[13] Dept. of Biochemistry and Molecular Genetics, Hospital Clínic, 08036 Barcelona, Spain
[14] Center for Biomedical Research Network in Gastrointestinal and Liver Diseases (CIBEREHD), Carlos III Health Institute, 28029 Madrid, Spain
[15] Institute for Medical Engineering & Science, Massachusetts Institute of Technology (MIT), Cambridge, MA 02139, USA
[16] Molecular Targets Program, Div. of Oncology, Dept. of Medicine, J.G. Brown Cancer Center, Louisville, KY 40202, USA
[17] ICREA, 08010 Barcelona, Spain

[#] and [¶]: these authors contributed equally to the work

* Address correspondence to: idib412@recerca.clinic.cat

Keywords: Atherosclerosis / Cholesterol efflux / Lipid Traffic / Macrophages / Macrophage-targeted Gene Delivery / Macrophage-targeted Nanoparticles

SUPPLEMENTARY FIGURES

Supplementary Figure S1

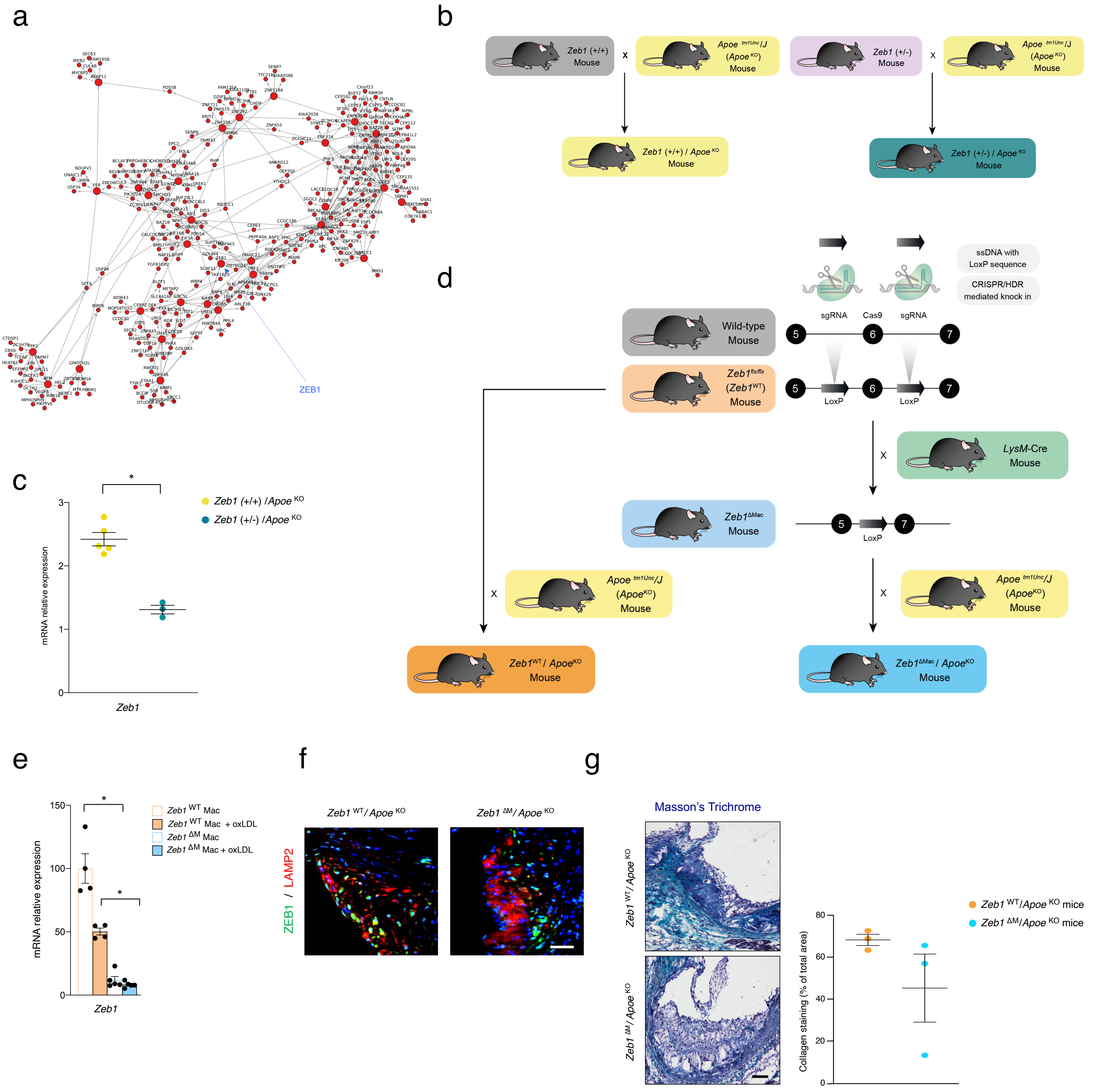

SUPPLEMENTARY FIGURE S1

ZEB1 expression in macrophages protects against atherosclerotic plaque formation. (a) ZEB1 is one of the 30 key drivers in the gene-regulatory coexpression networks identified by the STARNET database in the atherosclerotic aortic root of individuals with coronary artery disease versus the healthy aortic root of controls. **(b)** Schematic of the experimental mouse models *Zeb1*(+/+)/*Apoe*^{KO} and *Zeb1*(+/-)/*Apoe*^{KO} mice. **(c)** *Zeb1* mRNA levels in the peritoneal macrophages of *Zeb1*(+/+)/*Apoe*^{KO} and *Zeb1*(+/-)/*Apoe*^{KO} mice at the end of the Western feeding protocol. *Gapdh* was used as reference gene. (n=5,3) **(d)** Schematic of the generation of the experimental mice used in the study. The *Zeb1*^{flx} mouse was generated by CRISPR as described in Supplementary Information. *Zeb1*^{flx} mouse was then crossed with *Lysm*Cre to generate *Zeb1*^{WT} and *Zeb1*$^{\Delta M}$ mice, and then with the *Apoe*^{KO} mouse to generate the *Zeb1*^{WT}/*Apoe*^{KO} and *Zeb1*$^{\Delta M}$/*Apoe*^{KO} experimental mouse models. **(e)** *Zeb1* mRNA levels in the peritoneal macrophages of *Zeb1*^{WT} and *Zeb1*$^{\Delta M}$ macrophages in the presence or absence of oxLDL. (n=4,4,5,5) **(f)** Representative images of ZEB1 (HPA027524, 1/500, in green) and LAMP2/MAC-3 (M3/84, 1/50 in red) staining of aortic root sections from *Zeb1*^{WT}/*Apoe*^{KO} and *Zeb1*$^{\Delta M}$/*Apoe*^{KO} mice at the end of Western diet feeding protocol. Scale bar: 50 µm. (n=3) **(g)** As in Fig. 1k, but collagen was assessed using Masson's Trichrome. Representative images and quantification. Scale bar: 100 µm. (n=3). Graphs represent mean values +/- SEM with two-tailed unpaired Mann-Whitney test. $p \leq 0.001$ (***), $p \leq 0.01$ (**) or $p \leq 0.05$ (*) levels, or non-significant (ns) for values of $p > 0.05$. Raw data along with p values from statistical analyses are included in the Source Data file.

Supplementary Figure S2

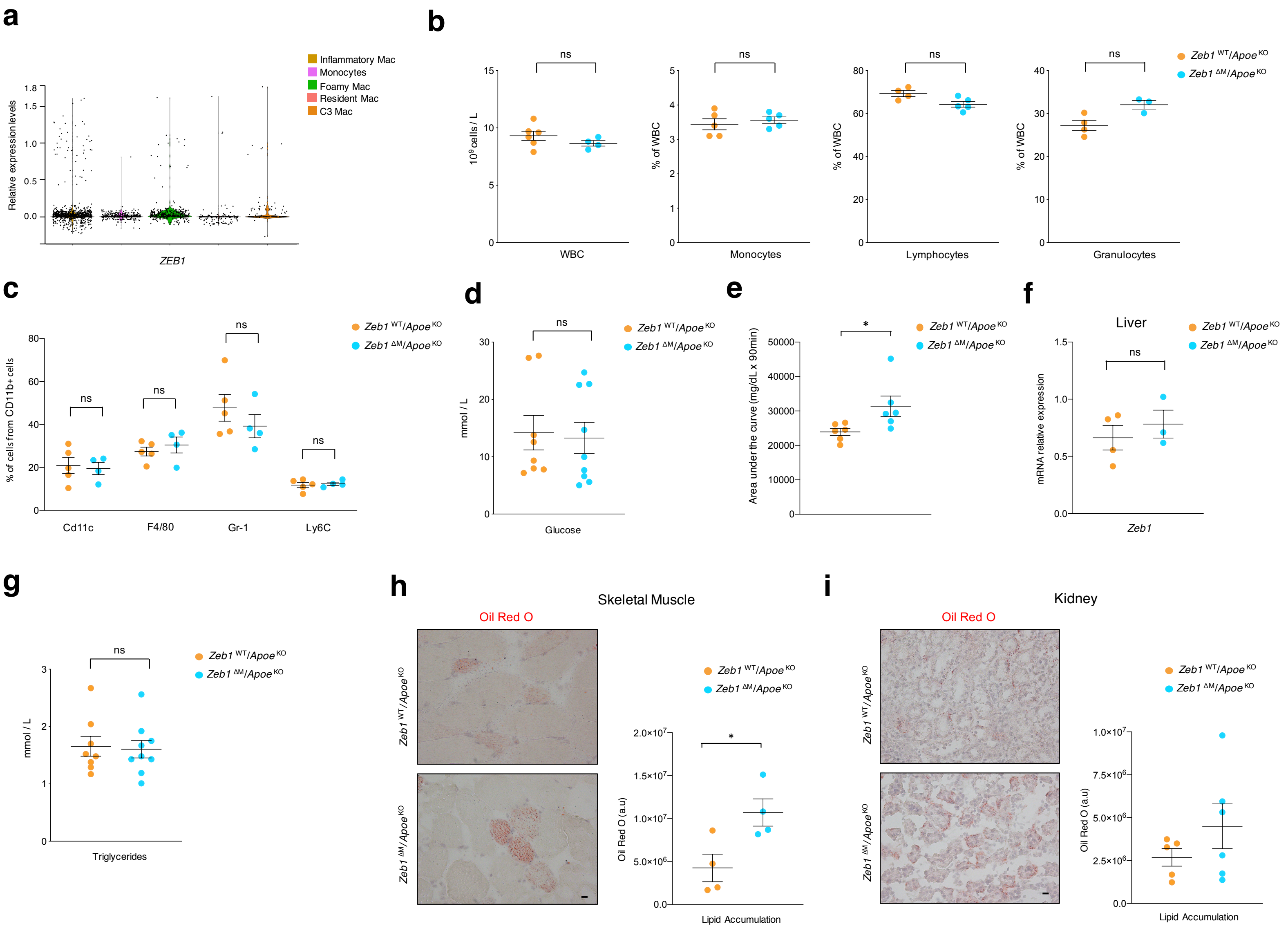

SUPPLEMENTARY FIGURE S2

***Zeb1*$^{\Delta M}$/*Apoe*^{KO} mice exhibit systemic inflammation and fat accumulation. (a)** Analysis of macrophage subpopulations in three scRNAseq datasets of human atherosclerotic coronary plaques as described in Supplementary Materials and Methods. **(b)** Total white blood cells (WBC) (n=6,4), monocytes (n=5), lymphocytes (n=4,5), and granulocytes (n=4,3) in *Zeb1*^{WT}/*Apoe*^{KO} and *Zeb1*$^{\Delta M}$/*Apoe*^{KO} mice at the end of the Western diet protocol were quantified as described in Supplementary Materials and Methods. **(c)** The percentage of CD11c^{+}, F4/80^{+}, Gr1^{+}, and Ly6C^{+} among CD11b^{+} cells from the spleens of *Zeb1*^{WT}/*Apoe*^{KO} and *Zeb1*$^{\Delta M}$/*Apoe*^{KO} mice were analyzed by FACS. (n=5,4). **(d)** Serum levels of glucose were determined in *Zeb1*^{WT}/*Apoe*^{KO} (n=8) and *Zeb1*$^{\Delta M}$/*Apoe*^{KO} (n=9) mice fed with the Western diet protocol. **(e)** Area under the curve calculated from Figure 2o. (n=6). **(f)** *Zeb1* mRNA levels in liver macrophages (CD45^{+} CD11b^{+} and F4/80^{+}) of *Zeb1*^{WT}/*Apoe*^{KO} (n=4) and *Zeb1*$^{\Delta M}$/*Apoe*^{KO} (n=3) mice fed with the Western diet protocol. **(g)** As in Supplementary Fig. S2d but triglyceride were determined (n=8,9). **(h)** Assessment of lipid accumulation in the gastrocnemius of *Zeb1*^{WT}/*Apoe*^{KO} and *Zeb1*$^{\Delta M}$/*Apoe*^{KO} mice by staining with ORO. Representative images and quantification. Scale bar: 20 µm. (n=4). **(i)** As in (h), but ORO staining of kidneys (n= 5,6). Scale bar: 20 µm. Graphs represent mean values +/- SEM with two-tailed unpaired t-test. $p \leq 0.001$ (***), $p \leq 0.01$ (**) or $p \leq 0.05$ (*) levels, or non-significant (ns) for values of $p > 0.05$. Raw data along with p values from statistical analyses are included in the Source Data file.

Supplementary Figure S3

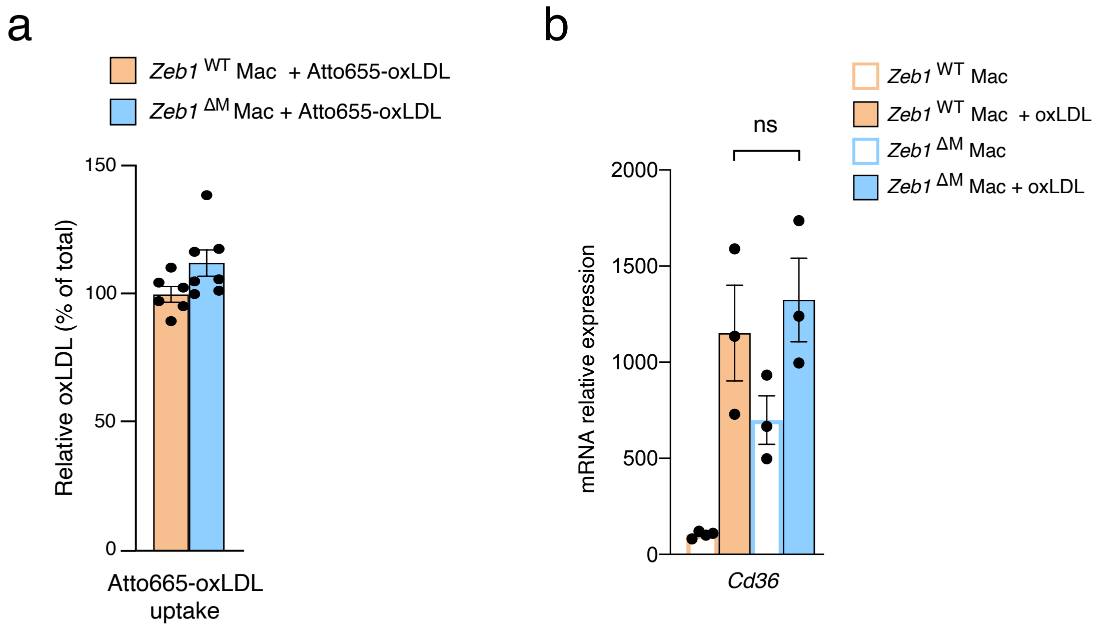

SUPPLEMENTARY FIGURE S3

ZEB1 inhibits lipid accumulation in macrophages by inhibiting cholesterol efflux. **(a)** Uptake of Atto655-oxLDL (excitation peak: 662 nm; emission peak: 680 nm) by *Zeb1*^{WT} and *Zeb1*$^{\Delta M}$ macrophages (n=6,7). **(b)** Relative mRNA levels of *Cd36/Scarb3* in *Zeb1*^{WT} and *Zeb1*$^{\Delta M}$ macrophages in the presence or absence of 50 μg/mL of oxLDL for 24 h. (n=4,3,3,3). Graphs represent mean values +/- SEM with two-tailed unpaired t-test. $p \leq 0.001$ (***), $p \leq 0.01$ (**) or $p \leq 0.05$ (*) levels, or non-significant (ns) for values of $p > 0.05$. Raw data along with p values from statistical analyses are included in the Source Data file.

Supplementary Figure S4

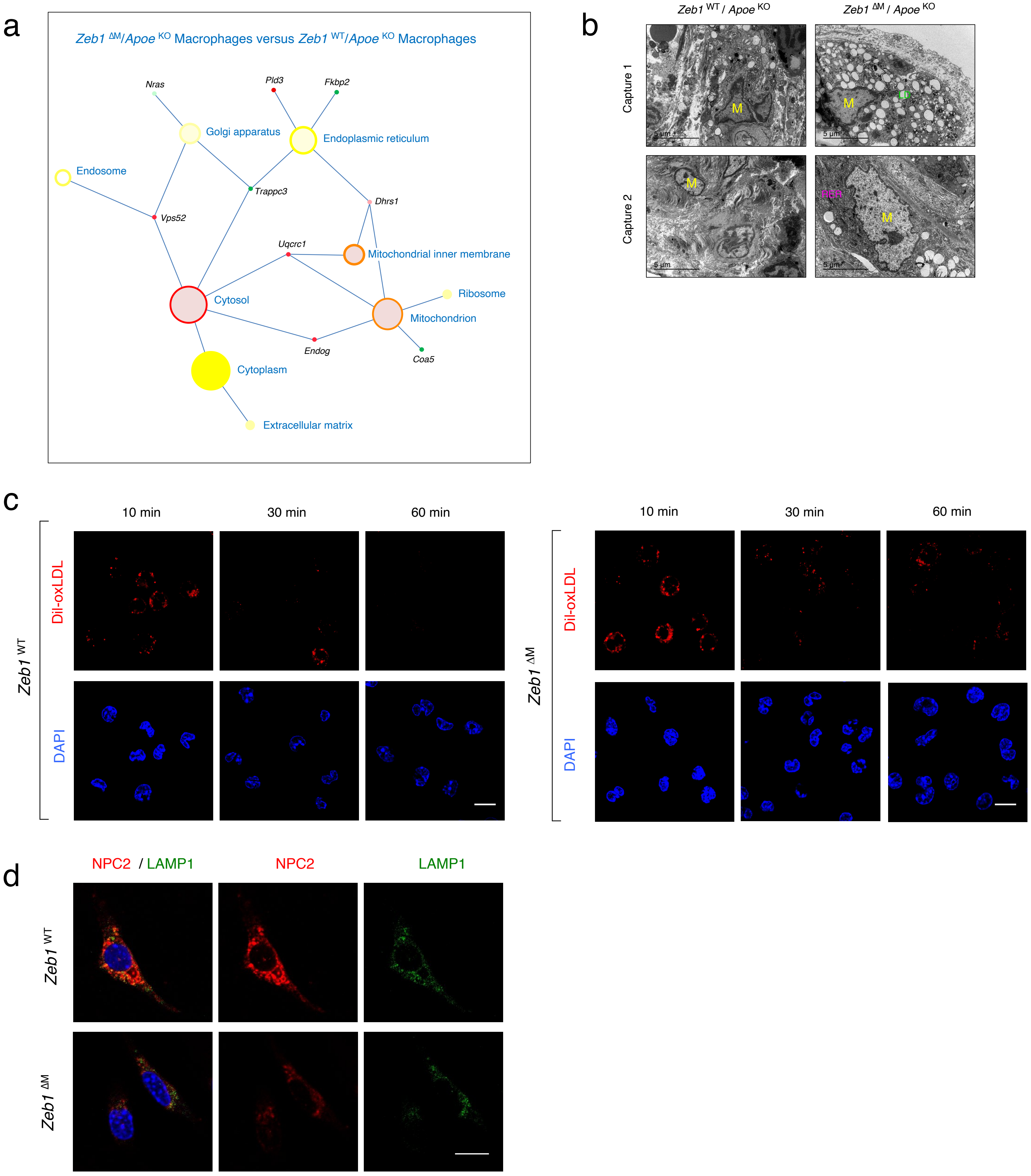

SUPPLEMENTARY FIGURE S4

***Zeb1* deletion alters intracellular lipid traffic in macrophages.** **(a)** Gene network analysis of the RNAseq of infiltrating macrophages in the plaque of *Zeb1*^{WT}/*Apoe*^{KO} and *Zeb1*$^{\Delta M}$/*Apoe*^{KO} mice at the end of the Western diet feeding protocol was conducted with NetworkAnalyst (https://www.networkanalyst.ca). **(b)** As in Fig. 4c, the ultrastructure of the atherosclerotic plaque in *Zeb1*^{WT}/*Apoe*^{KO} and *Zeb1*$^{\Delta M}$/*Apoe*^{KO} mice. Two captures for each genotype are shown. RER: rough endoplasmic reticulum; LD: lipid droplets; M: monocyte; Scale bar: 5 µm. **(c)** Single staining pictures of Fig. 4f. Scale bar: 10 µm. **(d)** Single staining pictures of Fig. 4h. Scale bar: 10 µm.

Supplementary Figure S5

a

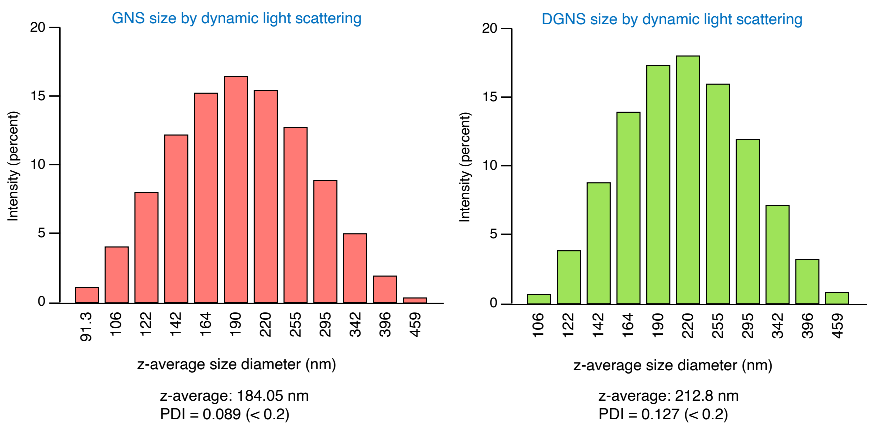

	GNS	DGNS	DGNS-Ctrl	DGNS-*Zeb1*
**Zeta potential** (mV)	– 34.3	45.7	– 47.4	– 32.0
**Osmolality** (mOsmol/Kg)	287 ± 2	301 ± 14	280 ± 18	285 ± 21

b

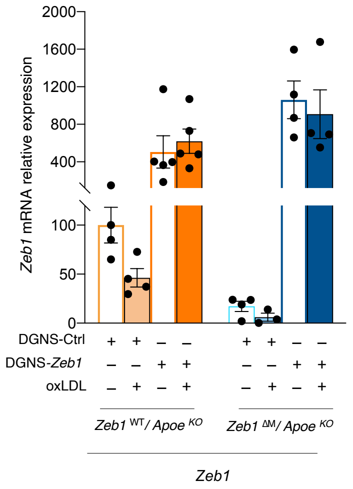

SUPPLEMENTARY FIGURE S5

Synthesis and characterization of nanoparticles. (a) Characterization of nanoparticles. Physicochemical characterization of GNS, DGNS, DGNS-Ctrl, DGNS-*Zeb1*. Zeta average (z-average) size distribution and polydispersity index (PDI) of GNS and DGNS were measured using dynamic light scattering. Zeta potential (mV) and osmolarity of GNS, DGNS, DGNS-Ctrl, and DGNS-*Zeb1*. (**b)** Peritoneal macrophages from *Zeb1*^{WT} and *Zeb1*$^{\Delta M}$ mice were incubated for 72 h with 100 ng/mL of either DGNS-Ctrl or DGNS-*Zeb1* and in the presence or absence of 50 µg/mL of oxLDL during the last 24 h and the expression of *Zeb1* assessed by qRT-PCR (n=4,4,5,5,4,3,4,4). Graphs represent mean values +/- SEM. Raw data along with p values from statistical analyses are included in the Source Data file.

Supplementary Figure S6

a

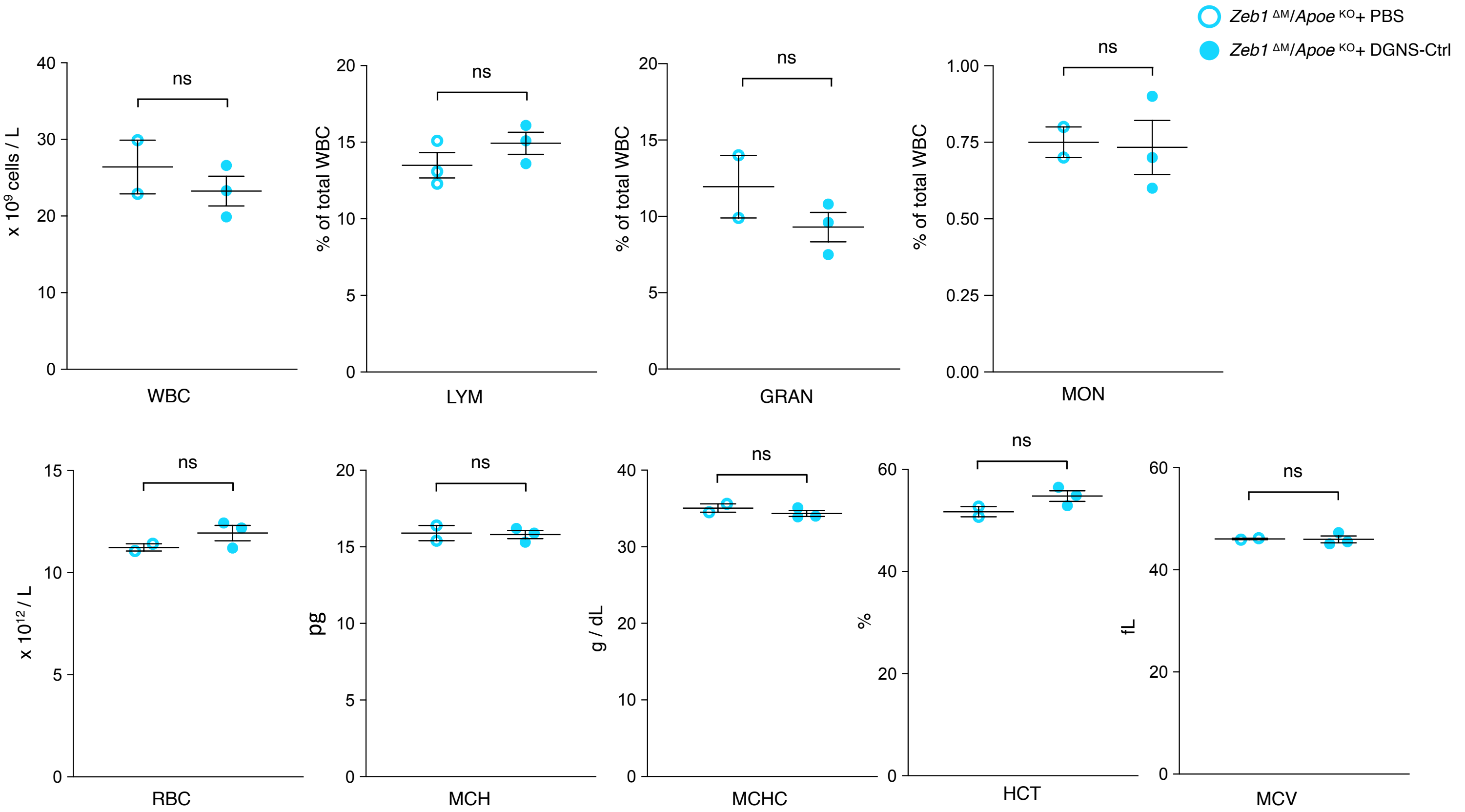

b

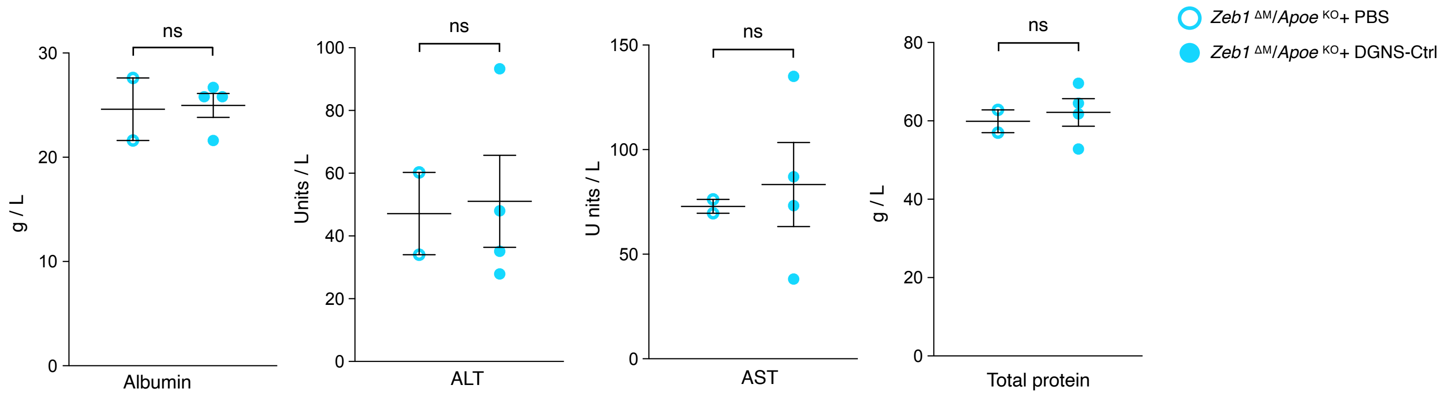

c

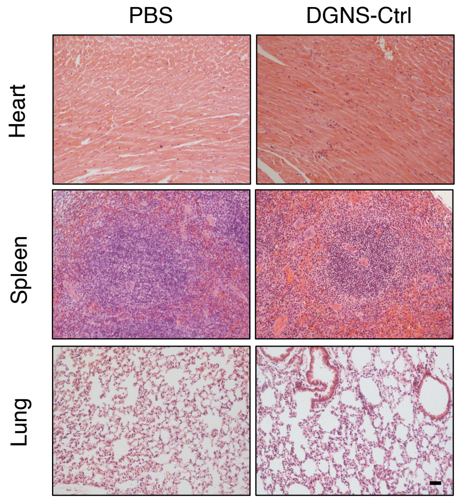

SUPPLEMENTARY FIGURE S6

***In vivo* use of macrophage-targeted nanoparticles expressing ZEB1 reduce atherosclerotic plaque formation. (a)** Hematological parameters of *Zeb1*$^{\Delta M}$/*Apoe*^{KO} mice treated with DGNS-Ctrl or DGNS-*Zeb1* as in Fig. 6a. White blood cells (WBC), lymphocytes (LYM), Granulocytes (GRAN), monocytes (MON), red blood cells (RBC), mean corpuscular hemoglobin (MCH), mean corpuscular hemoglobin concentration (MCHC), hematocrit (HCT), mean corpuscular volume (MCV). (n=2,3) **(b)** As in (a), biochemical parameters in serum. Albumin, Alanine transaminase (ALT), Aspartate transaminase (AST), and total protein. (n=2,4) **(c)** As in Fig. 6b, but pictures of the heart, spleen, and lung. Scale bar: 20 µm. (n=2). Graphs represent mean values +/- SEM with two-tailed unpaired Mann-Whitney test. $p \leq 0.001$ (***), $p \leq 0.01$ (**) or $p \leq 0.05$ (*) levels, or non-significant (ns) for values of $p > 0.05$. Raw data along with p values from statistical analyses are included in the Source Data file.

Supplementary Figure S7

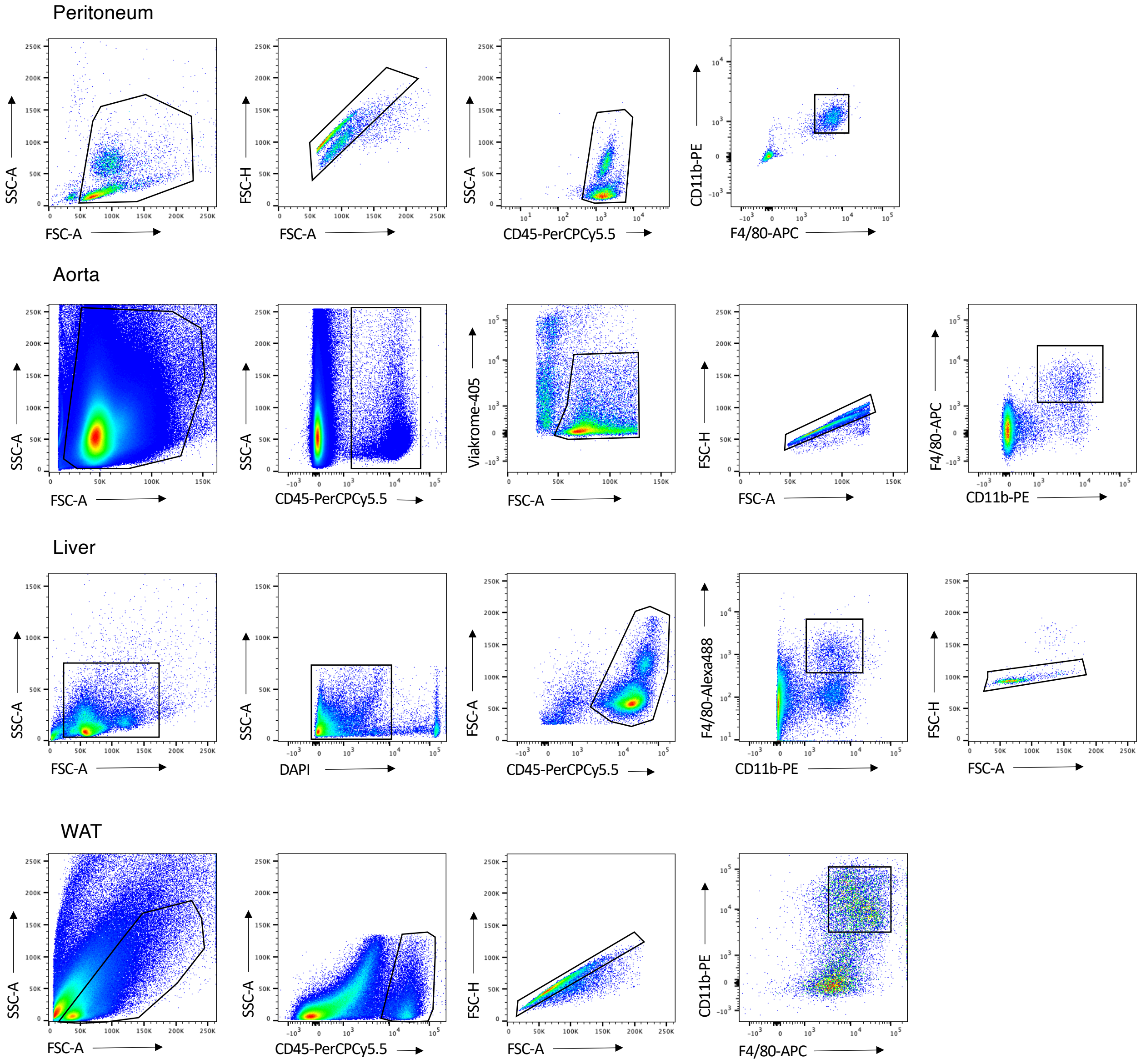

<u>SUPPLEMENTARY FIGURE S7</u>

Gating strategy for peritoneal, aorta, liver and white adipose tissue macrophages.

SUPPLEMENTARY TABLES

Supplementary Table S1: Basic Epidemiological and Clinical Data of Patients Undergoing Endarterectomies (n = 24)

Epidemiological and clinical characteristics	Values
Sex	
Male	86.6%
Female	13.3%
Age (years)	69.6 (55-81)
Cerebrovascular Accidents	
Asymptomatic	53.3%
Transient Ischemic Attack	23.3%
Stroke	23.3%
Comorbidities and other characteristics	
Dyslipidemia	56.6%
Diabetes	36.6%
Hipertension	66.6%
Coronary Artery Disease	26.6%
Smoking	
Smoker	53.3%
Ex-smoker	33.3%
Non-smoker	13,3%
Type of plaque	
Ulcerated-complicated	20.0%
Ulcerated	20.0%
Fibrous-complicated	26.6%
Fibrous	10.0%
Preatheroma	23.3%
Intra-plaque angiogenesis	
Yes	36.6%
No	63.3%

Supplementary Table S2: sgRNA and ssDNA oligonucleotides used in the generation of the *Zeb1*$^{fl/fl}$ mouse *

sgRNA oligonucleotides	Sequence
sgRNA 5’	5’-TTACAGACACCTCTAACACAAGG-3’
sgRNA 3’	3’-AGTACCAGCAAACCCTTTCTTGG-5’
**sgRNA oligonucleotides**	**Oligos for cloning into the px330 vector**
sgRNA 5’	Forward: CACCgttacagacacctctaacaca<br>Reverse: AAACtgtgttagaggtgtctgtaaC
sgRNA 3’	Forward: CACCgagtaccagcaaaccctttct<br>Reverse: AAACagaaagggtttgctggtactC
**ssDNA oligonucleotides**	**Sequence**
ssDNA #1	5’- agctaagtcccttcaagtgcctggtcactgaggaaagctggggTTACAGACACCTCTAAC GCTAGCataacttcgtatagcatacattatacgaagttatACAAGGcttcctccccaaagggagccgtaca gacatgaaaatatttatcaatcaaaggc - 3’
ssDNA #2	3’- aaccaaaggttaacctaactcctaacaaaggagttggcacacgaAGTACCAGCAAACCCTGA ATTCataacttcgtataatgtatgctatacgaagttatTTCTTGGctttatggtgaatgggaacatggttgtttaat agtgatcataagcaaagaaga - 5’

* The code for colored bases is as follows: blue bases refer to the sgRNA sequence, red bases refer to the protospacer adjacent motif, bases in orange refer to the LoxP sequences while bases in green indicate the restriction enzyme target. Underlined bases correspond to the homology arms.

Supplementary Table S3: Primary and Secondary Antibodies

Unconjugated Primary Antibodies		
**Target protein**	**Source**	**Clone (Catalog Number)**
GAPDH	Proteintech	1E6D9 (60004-1-Ig)
LAMP1 (CD107a)	Sigma-Aldrich	H4A3 (MABC1108)
LAMP2 (CD107b/MAC-3)	Santa Cruz Biotechnology	M3/84 (sc-19991)
NPC2	Proteintech	19888-1-AP
SREBP1c	Novus Biologicals	2A4 (NB600)
ZEB1	Sigma-Aldrich	HPA027524
**Conjugated Primary Antibodies**		
**Target protein**	**Source**	**Clone (Catalog Number)**
CD9: anti-mouse CD9 conjugated APC	BioLegend	MZ3 (124811)
CD11b: anti-mouse CD11b PE-conjugated	ImmunoTools GmbH	M1/70.15 (22159114)
CD11c: anti-mouse CD11c conjugated PE-Cy7	Biolegend	N418 (117317)
CD45: anti-mouse CD45-conjugated PerCP/Cy5.5	BioLegend	30-F11 (103132)
CD86: anti-mouse CD86 conjugated BV421	BioLegend	GL-1 (105031)
F4/80: Alexa Fluor® 488 anti-mouse F4/80	BioLegend	BM8 (123119)
F4/80: APC anti-mouse F4/80	BioLegend	BM8 (123115)
GR1: anti-mouse Gr-1 conjugated FITC	Immunotools GmbH	RB6-8C5 (22155243)
Ly6C: anti-mouse Ly6C conjugated PerCP/Cy5.5	eBioscience	HK1.4 (45-5932-80)
**Secondary Antibodies**		
Anti-Mouse: Alexa Fluor® 488 AffiniPure Donkey Anti-Mouse IgG (H+L)	Jackson ImmunoResearch	715-545-150
Anti-Mouse: Peroxidase-AffiniPure Donkey anti-Mouse IgG (H+L)	Jackson ImmunoResearch	715-035-151
Anti-Rabbit: Alexa Fluor® 488 AffiniPure Donkey Anti-Rabbit IgG (H+L)	Jackson ImmunoResearch	711-545-152
Anti-Rat: Rhodamine Red™-X (RRX) AffiniPure Donkey Anti-Rat IgG (H+L)	Jackson ImmunoResearch	712-295-150
Anti-Rabbit: Rhodamine Red™-X (RRX) AffiniPure Donkey Anti-Rabbit IgG (H+L)	Jackson ImmunoResearch	711-295-152
**Normal sera**		
Donkey: Normal Serum	Jackson ImmunoResearch	017-000-121
Mouse: Gamma Globulin	Jackson ImmunoResearch	015-000-002

<u>Supplementary Table S4</u>: DNA primers used in qRT-PCR

**Human Genes**	**Forward 5'- 3'**	**Reverse 3'- 5'**
GAPDH	TGCACCACCAACTGCTTAGC	GGCATGGACTGTGGTCATGAG
ZEB1	AGCAGTGAAAGAGAAGGGAATGC	GGTCCTCTTCAGGTGCCTCAG
**Mouse Genes**	**Forward 5'- 3'**	**Reverse 3'- 5'**
Abca1	CAGGGTGGCTCTTCTCATCAAT	GCCGTCTTTCCAGGACAGTATG
Abcg1	TCCGGATTCTTTGTCAGCTT	CAGGACGATGAAATCCAGGT
Adss1	ACCGATGGTGAAAGATGGAG	TGCCCAAACCTGTACAAACA
Asl	GTGGAATGTGGATGTGCAGG	GTCCTGTGTGTAGCTTCCCT
Chmp1b	TGAAATTCGCGGCCAAAGAAC	ACTTCCATGTTGCCCTTCTGA
Gapdh	CGACTTCAACAGCAACTCCCACTCTTCC	TGGGTGGTCCAGGGTTTCTTACTCCTT
Nr1h3	GCAGGACCAGCTCCAAGTAG	GGCTCACCAGCTTCATTAGC
Ppargc1a	TTGCTAGCGGTTCTCACAGA	TAAGACCGCTGCATTCATTG
Prkaa1	CTTGACGTGGTGGGAAAAAT	ATAATCAAATAGCTCTCCTCCAGA
Rpl19	GCATCCTCATGGAGCACAT	CTGGTCAGCCAGGAGCTT
Slc2a1	GTCCTGCTGCTATTGCTGTG	CCTCGGGTGTCTTGTCATT
Slc2a4	GGTTATCAATGCCCCACAGA	AGCATAGCCCTTTTCCTTCC
Vps52	GGAAGTCGGAAGTGAAGCTG	AGGTTTGCCTGGATGTGAAC
Zeb1	ATTCCCCAAGTGGCATATACA	GAGCTAGTGTCTTGTCTTTCATCC
