# Supplementary material for: Atherosclerotic plaque development in mice is enhanced by myeloid ZEB1 downregulation
Source: Nat Commun. 2023 Dec 14;14:8316. doi: 10.1038/s41467-023-43896-7 (PMC10721632; doi:10.1038/s41467-023-43896-7)
Supplement: Supplementary file 3 — Description of Additional Supplementary Files [file 41467_2023_43896_MOESM3_ESM.pdf]

Description of Additional Supplementary Files

Supplementary Movie 1 : (pHrodoTM Green LDL-*Zeb1*^{WT})

The uptake and transport of pHrodoTM Green-LDL fluorescence in *Zeb1*^{WT} macrophages was captured over time (0-30 min) by confocal microscopy. Representative video.

Supplementary Movie 2 : (pHrodoTM Green LDL- *Zeb1*^{1M})

The uptake and transport of pHrodoTM Green-LDL fluorescence in *Zeb1*^{1M} macrophages was captured over time (0-30 min) by confocal microscopy. Representative video.
