# Supplementary material for: Atherosclerotic plaque development in mice is enhanced by myeloid ZEB1 downregulation
Source: Nat Commun. 2023 Dec 14;14:8316. doi: 10.1038/s41467-023-43896-7 (PMC10721632; doi:10.1038/s41467-023-43896-7)
Supplement: Supplementary file 6 — Reporting Summary [file 41467_2023_43896_MOESM6_ESM.pdf]

nature portfolio

Corresponding author(s): NCOMMS-23-03596A

Last updated by author(s): 11/21/2023

Reporting Summary

Nature Portfolio wishes to improve the reproducibility of the work that we publish. This form provides structure for consistency and transparency in reporting. For further information on Nature Portfolio policies, see our Editorial Policies and the Editorial Policy Checklist.

Statistics

For all statistical analyses, confirm that the following items are present in the figure legend, table legend, main text, or Methods section.

n/a	Confirmed	
☐	☒	The exact sample size (n) for each experimental group/condition, given as a discrete number and unit of measurement
☐	☒	A statement on whether measurements were taken from distinct samples or whether the same sample was measured repeatedly
☐	☒	The statistical test(s) used AND whether they are one- or two-sided<br>*Only common tests should be described solely by name; describe more complex techniques in the Methods section.*
☒	☐	A description of all covariates tested
☒	☐	A description of any assumptions or corrections, such as tests of normality and adjustment for multiple comparisons
☐	☒	A full description of the statistical parameters including central tendency (e.g. means) or other basic estimates (e.g. regression coefficient) AND variation (e.g. standard deviation) or associated estimates of uncertainty (e.g. confidence intervals)
☐	☒	For null hypothesis testing, the test statistic (e.g. F, t, r) with confidence intervals, effect sizes, degrees of freedom and P value noted<br>*Give P values as exact values whenever suitable.*
☒	☐	For Bayesian analysis, information on the choice of priors and Markov chain Monte Carlo settings
☒	☐	For hierarchical and complex designs, identification of the appropriate level for tests and full reporting of outcomes
☒	☐	Estimates of effect sizes (e.g. Cohen's d, Pearson's r), indicating how they were calculated

Our web collection on statistics for biologists contains articles on many of the points above.

Software and code

Policy information about availability of computer code

Data collection	FACS data was analyzed using a BD FACSCanto, a BD FACSFortessa analyzer or a BD FACSARIA SORP cell sorter.<br>qPCR data was quantified in a LightCycler 96 Roche Instrument.<br>Western Blot membranes were revealed in a ChemiDoc Biorad imaging system.<br>ELISA data was measured in a TECAN Infinite M Nano.<br>IF images were captured using a inverted Leica AF6000 microscope or a Zeiss Axiovert 100 inverted microscope.<br>Tritium radiactivity was measured in a Hidex sense beta microplate reader.<br>A JEOL JEM-1010 transmission electron microscope was used to pictured ultrastructure of cells and tissues.
Data analysis	For FACS data analysis was used FlowJo 10.8.1.<br>To plot the data and for statistical analysis was used GraphPad Prism 8.4.3.<br>To quantification of images and IF composite was used ImageJ 2.1.0.<br>Rayplex Array and ELISA data were analyzed using GainData Arigo's ELISA calculator.<br>To analyze qPCR data was used the LightCycler 96 SW 1.1 program.

For manuscripts utilizing custom algorithms or software that are central to the research but not yet described in published literature, software must be made available to editors and reviewers. We strongly encourage code deposition in a community repository (e.g. GitHub). See the Nature Portfolio guidelines for submitting code & software for further information.

Data

Policy information about availability of data

All manuscripts must include a data availability statement. This statement should provide the following information, where applicable:

- Accession codes, unique identifiers, or web links for publicly available datasets
- A description of any restrictions on data availability
- For clinical datasets or third party data, please ensure that the statement adheres to our policy

The RNAseq dataset has been submitted to the Gene Expression Omnibus (GEO) database and assigned accession number GSE206477 (https://www.ncbi.nlm.nih.gov/geo/query/acc.cgi?acc=GSE206477). Datasets of published RNAseq of human endarterectomies were obtained from GSE41571 (68) (https://www.ncbi.nlm.nih.gov/geo/query/acc.cgi?acc=GSE41571) and GSE163154 (69) (https://www.ncbi.nlm.nih.gov/geo/query/acc.cgi?acc=GSE163154). Datasets of single cell RNAseq were obtained from GSE116240 (3), https://www.ncbi.nlm.nih.gov/geo/query/acc.cgi?acc=GSE116240; GSE149070 (4), https://www.ncbi.nlm.nih.gov/geo/query/acc.cgi?acc=GSE149070; (5) https://figshare.com/s/c00d88b1b25ef0c5c788 and GSE131778 (6), https://www.ncbi.nlm.nih.gov/geo/query/acc.cgi?acc=GSE131778. Source data are provided with this paper.

Research involving human participants, their data, or biological material

Policy information about studies with human participants or human data. See also policy information about sex, gender (identity/presentation), and sexual orientation and race, ethnicity and racism.

Reporting on sex and gender	The sex characteristics of patients are detailed in Supplementary Table S1.
Reporting on race, ethnicity, or other socially relevant groupings	The population characteristics of patients are detailed in Supplementary Table S1.
Population characteristics	The patients were aged between 55 to 81 and were classified depending on whether or not they had suffered a cerebrovascular accident. Other characteristics that were recorded are dyslipidemia (56.6%), diabetes (36.6), hypertension (66.6%), coronary artery disease (26.6%), smoking, type of plaque and intra-plaque angiogenesis. More details of population characteristics are in Supplementary Table S1.
Recruitment	Samples of atheroma plaques were obtained from patients, aged 55 to 81, subjected to programmed endarterectomy. A majority of the patients had suffered from cerebrovascular accident previus to endarterectomy. Key data recorded from all samples included the degree, the presence of bilateral pathology, morphological characteristics , plaque stability, an the assessment of angiogenesis within the plaque. The samples were classified in two cohorts: symptomatic or asymptomatic dependent on whether or not they had suffered a cerebrovascular accident, grade IV to VI as previously reported (Hurtado et al., 2011). To classify the type of stroke, it was used the oxfordshire classification and it was futher confirmed through nuclear magnetic resonance imaging. The samples were processed following standardized biobank protocol without delay in processing.
Ethics oversight	The use of human samples was approved by the local Ethics Committee at the Hospital of Bellvitge under protocol 14/04. All human samples were obtained with the written informed consent of patients, and conformed with the principles of the Helsinki Declaration.

Note that full information on the approval of the study protocol must also be provided in the manuscript.

Field-specific reporting

Please select the one below that is the best fit for your research. If you are not sure, read the appropriate sections before making your selection.

☒ Life sciences ☐ Behavioural & social sciences ☐ Ecological, evolutionary & environmental sciences

For a reference copy of the document with all sections, see nature.com/documents/nr-reporting-summary-flat.pdf

Life sciences study design

All studies must disclose on these points even when the disclosure is negative.

Sample size	The sample size in each experiment is detailed in the figure legend and represented as data point in each graph. We did not perform a formal sample size calculation prior to the start of the study. Our choice of sample size was guided by a combination of prior knowledge, ethical considerations, and based on previous studies (Nat Commun 13:215 and Nat Commun 14:4622). Also, we aimed to maximize the use of animals to ethical principles, as well as the principle of the 3 Rs (replacement, reduction, refinement) to minimize the number of animals. In the allocation of groups, we took care to achieve a balanced distribution. In in vitro assays, where variability tends to be lower, we employed fewer biological replicates. Each experiment was replicated at least twice, bolstering the robustness of our findings.
Data exclusions	No data were excluded.
Replication	The experiments were carried out at least in 2-3 independent experiment and the results were consistent between experiments.

Randomization	The allocated of the mice was random. Mice of each genotype were randomly assigned to treatment or control groups and they were process in paralel. To minimize cage effects, mice were housed in mixed cages.
Blinding	In our RNAseq analysis, we followed a blinded approach. Specialized analysts carried out the analysis without any knowledge of the identity of the samples. However, in the rest of the experiments, blinding was not possible in practice since the coexistence of the transgenic mice in the same cage means that they have to have identification marks to distinguish the mice. Mice of each genotype were randomly assigned to treatment or control groups. Furthermore, data collection and analysis were quantitative rather than qualitative.

Reporting for specific materials, systems and methods

We require information from authors about some types of materials, experimental systems and methods used in many studies. Here, indicate whether each material, system or method listed is relevant to your study. If you are not sure if a list item applies to your research, read the appropriate section before selecting a response.

Materials & experimental systems

n/a	Involved in the study
☐	☒ Antibodies
☒	☐ Eukaryotic cell lines
☒	☐ Palaeontology and archaeology
☐	☒ Animals and other organisms
☒	☐ Clinical data
☒	☐ Dual use research of concern
☒	☐ Plants

Methods

n/a	Involved in the study
☒	☐ ChIP-seq
☐	☒ Flow cytometry
☒	☐ MRI-based neuroimaging

Antibodies

Antibodies used	Primary and secondary antibodies used in the study are listed in Supplementary Table S4<br>Primary unconjugated antibodies:<br><br>GAPDH Proteintech 1E6D9 (60004-1-Ig)<br>LAMP1 (CD107a) Sigma-Aldrich H4A3 (MABC1108)<br>LAMP2 (CD107b/MAC-3) Santa Cruz Biotechnology M3/84 (sc-19991)<br>NPC2 Proteintech 19888-1-AP<br>SREBP1c Novus Biologicals 2A4 (NB600)<br>ZEB1 Sigma-Aldrich HPA027524<br><br>Conjugated antibodies:<br><br>CD9: anti-mouse CD9 conjugated APC BioLegend MZ3 (124811)<br>CD11b: anti-mouse CD11b PE-conjugated ImmunoTools GmbH M1/70.15 (22159114)<br>CD11c: anti-mouse CD11c conjugated PE-Cy7 Biolegend N418 (117317)<br>CD45: anti-mouse CD45-conjugated PerCP/Cy5.5 BioLegend 30-F11 (103132)<br>CD86: anti-mouse CD86 conjugated BV421 BioLegend GL-1 (105031)<br>F4/80: Alexa Fluor® 488 anti-mouse F4/80 BioLegend BM8 (123119)<br>F4/80: APC anti-mouse F4/80 BioLegend BM8 (123115)<br>GR1: anti-mouse Gr-1 conjugated FITC Immunotools GmbH RB6-8C5 (22155243)<br>Ly6C: anti-mouse Ly6C conjugated PerCP/Cy5.5 HK1.4 (45-5932-80)
Validation	The antibodies used in the study were validated by the corresponding manufacturer and/or were widely used in the literature. Commercial antibodies were employed in accordance with the manufacturers' recommendations as provided on their datasheets and official websites.<br><br>GAPDH Proteintech 1E6D9 (60004-1-Ig) https://www.ptglab.com/products/GAPDH-Antibody-60004-1-Ig.htm (8830 citations)<br>LAMP1 (CD107a) Sigma-Aldrich H4A3 (MABC1108) https://www.sigmaaldrich.com/ES/es/product/mm/mabc1108<br>LAMP2 (CD107b/MAC-3) Santa Cruz Biotechnology M3/84 (sc-19991) https://www.scbt.com/p/lamp-2-antibody-m3-84 (54 citations)<br>NPC2 Proteintech 19888-1-AP https://www.ptglab.com/products/NPC2-Antibody-19888-1-AP.htm (6 citations)<br>SREBP1c Novus Biologicals 2A4 (NB600) https://www.novusbio.com/products/srebp1-antibody-2a4_nb600-582 (55 citations)<br>ZEB1 Sigma-Aldrich HPA027524 https://www.sigmaaldrich.com/ES/es/product/sigma/hpa027524 (120 citations)<br><br>CD9: anti-mouse CD9 conjugated APC BioLegend MZ3 (124811) https://www.biolegend.com/en-gb/products/apc-anti-mouse-cd9-antibody-16739?GroupID=BLG10528 (3 citations)<br>CD11b: anti-mouse CD11b PE-conjugated ImmunoTools GmbH M1/70.15 (22159114) http://www.immunotools.de/html/datas-pem/22159114.pdf (3 citations)<br>CD11c: anti-mouse CD11c conjugated PE-Cy7 Biolegend N418 (117317) https://www.biolegend.com/en-us/products/pe-cyanine7-anti-mouse-cd11c-antibody-3086?GroupID=BLG11937 (225 citations)<br>CD45: anti-mouse CD45-conjugated PerCP/Cy5.5 BioLegend 30-F11 (103132) https://www.biolegend.com/fr-lu/products/percpcyanine5-5-anti-mouse-cd45-antibody-4264?GroupID=BLG6829 (214 citations)<br>CD86: anti-mouse CD86 conjugated BV421 BioLegend GL-1 (105031) https://www.biolegend.com/en-gb/products/brilliant-

violet-421-anti-mouse-cd86-antibody-7282?GroupID=BLG10719 (17 citations)
F4/80: Alexa Fluor® 488 anti-mouse F4/80 BioLegend BM8 (123119) https://www.biolegend.com/en-us/products/alexa-fluor-488-anti-mouse-f4-80-antibody-4073?GroupID=BLG5319 (82 citations)
F4/80: APC anti-mouse F4/80 BioLegend BM8 (123115) https://www.biolegend.com/de-de/explore-new-products/apcanti-mouse-f4-80-antibody-4071 (402 citations)
GR1: anti-mouse Gr-1 conjugated FITC Immunotools GmbH RB6-8C5 (22155243) https://www.biolegend.com/fr-ch/products/fitc-anti-mousely-6g-ly-6c-gr-1-antibody-458?GroupID=BLG4876 (127 citations)
Ly6C: anti-mouse Ly6C conjugated PerCP/Cy5.5 HK1.4 (45-5932-80) https://www.thermofisher.com/antibody/product/Ly-6C-Antibody-clone-HK1-4-Monoclonal/45-5932-82 (65 citations)

Animals and other research organisms

Policy information about studies involving animals; ARRIVE guidelines recommended for reporting animal research, and Sex and Gender in Research

Laboratory animals	The conditional Zeb1 flox allele mouse (Zeb1fl/fl, herein referred to as Zeb1WT) used in the study was generated at the Transgenesis Unit, a joint facility of the Spanish National Research Council (CSIC) [National Biotechnology Center (CSIC-CNB), the Severo Ochoa's Molecular Biology Center (CSIC-CBMSO)] and the Autonomous University of Madrid (Madrid, Spain). Mice were crossed multiple times with twild type C57BL6/J mice to generate the Zeb1fl/+ mice. The Zeb1fl/fl (Zeb1WT) mouse (B6.B6CBA-Zeb1em1/cnbbm) was crossed with a mouse carrying the Cre recombinase selectively in myeloid cells under the control of the endogenous lysozyme 2 (Lyz2, also referred as LysM) promoter/enhancer (official name: B6.129P2-Lyz2tm1(cre)Ifo/J). Zeb1WT, and Zeb1ΔM mice were crossed with Apoe(-/-) (referred hereafter to as ApoeKO) mice (B6.129P2-Apoetm1Unc/J) from The Jackson Laboratory (Strain #:002052) (Bar Harbor, ME, USA) (Piedrahita et al., 1992) to generate the experimental mouse models used in the study, namely Zeb1WT/ApoeKO and Zeb1ΔM/ApoeKO mice. Male mice of 6-8 weeks old were used. All the mice were housed in a temperature-controlled room maintained at 21-22ºC with a 12-hour light/dark cycle. The mice had access to chow or western diet and water ad libitum. The mice were euthanized by carbon dioxide inhalation.
Wild animals	There were not used wild animals in the study.
Reporting on sex	The study utilized only male mice because, despite Western-fed ApoeKO female mice developing larger atherosclerotic plaques, males in atherosclerosis mouse models fed with Western diet exhibit higher levels of serum lipoproteins and triglycerides, gain more weight and, more importantly, display higher levels of inflammatory markers (J Physiol Pharmacol. 61:309-316; Front Immunol 28:537; Mol Metab. 6:845-853; Arterioscler Thromb Vasc Biol. 39:1588-1601). In fact, the inflammatory state in atherosclerosis mouse models is considered more relevant for predicting sex-specific outcomes in humans than plaque size (Stroke. 49:419-425; Nat Rev Dis Primers. 5:56).
Field-collected samples	There were not used field-collected samples in the study.
Ethics oversight	The use of mice in this study has been approved by the University of Barcelona School of Medicine's Animal Experimentation Ethics Research Committee and assigned reference 358/18.

Note that full information on the approval of the study protocol must also be provided in the manuscript.

Flow Cytometry

Plots

Confirm that:

☒ The axis labels state the marker and fluorochrome used (e.g. CD4-FITC).

☒ The axis scales are clearly visible. Include numbers along axes only for bottom left plot of group (a 'group' is an analysis of identical markers).

☒ All plots are contour plots with outliers or pseudocolor plots.

☒ A numerical value for number of cells or percentage (with statistics) is provided.

Methodology

Sample preparation	Cells were isolated from mice and resuspended in FACS buffer (PBS 1x, 3% FBS, 1mM EDTA) with mouse gamma globulin for 30 min at 4ºC. Then, cells were washed and stained with the corresponding antibody for 45-60 min at 4ºC. After the staining, cells were wash and analyzed. More detailed are provided in methods section.
Instrument	BD FACSCanto analyzer, BD LSRFortessa cell analyzer or BD FACSARIA SORP cell sorter.
Software	FlowJo 10.8.1
Cell population abundance	The percentage and abundance of each population is detailed in the main text and in methods section.
Gating strategy	The gating strategy is described in methods.

☒ Tick this box to confirm that a figure exemplifying the gating strategy is provided in the Supplementary Information.
